# Characterization of Extended-spectrum β-lactamase-producing Clinical Isolates of *Shigella flexneri*

**DOI:** 10.3329/jhpn.v31i3.16834

**Published:** 2013-09

**Authors:** Jharna Mandal, V. Sangeetha, Nivedithadivya, Ankita Das, Subhash Chandra Parija

**Affiliations:** Department of Microbiology, Jawaharlal Institute of Postgraduate Medical Education and Research (JIPMER), Puducherry, India

Sir,

Antimicrobial agents are the mainstay of therapy in severe shigellosis. Conventionally, the fluoroquinolones have been the main drugs of choice for the treatment of shigellosis. With the emergence of fluoroquinolone resistance among *Shigella* (1-5), the third-generation cephalosporins have been used for the treatment of severe shigellosis. However, recent reports of resistance to the third-generation cephalosporins from India (6-8) made the selection of antibiotics for empirical therapy, particularly in children, even more complicated. Here, we report the 2 *Shigella flexneri* isolates which were genotypically proven to produce extended-spectrum β-lactamase (ESBL).

The study was undertaken in the Department of Microbiology and Department of Pediatrics, Jawaharlal Institute of Postgraduate Medical Education and Research (JIPMER), Puducherry, India. The three strains described in this study were isolated from the stool samples of paediatric patients coming to JIPMER hospital. One of the strains was isolated from a one-year old child with the manifestations of severe dysentery which had recurred more than three times while the second strain was isolated from a 10-month old infant who had been diagnosed to have acute gastroenteritis mimicking cholera. The stool samples from these children were plated directly onto MacConkey agar, Deoxycholate citrate agar, and Xylose lysine deoxycholate agar as well as into Selenite F broth for enrichment, which was subcultured after 18 hours in the abovementioned plates. The plates were incubated at 37 °C overnight under aerobic conditions. Following incubation, non-lactose-fermenting pale translucent colonies on MacConkey agar and Deoxycholate citrate agar (Himedia, Mumbai, India) and pink translucent colonies on Xylose lysine deoxycholate (Himedia, Mumbai, India) were selected for the biochemical tests (9). Further confirmation was done by slide agglutination using specific antisera (Denka-Seiken, Tokyo, Japan). Antibiotic susceptibility testing was done by Kirby Bauer method as per Clinical Laboratory Standards Institute (10) against ampicillin (A) 10 μg, ceftriaxone (Ci) 30 μg, ciprofloxacin (Cf) 5 μg, nalidixic acid (Na) 30 μg, furoxone (Fx) 300 μg, chloramphenicol (C) 30 μg, and cotrimoxazole (Co) 25 μg, which showed that the two strains were resistant to Ci. The MIC for ceftriaxone was determined by agar dilution method and E-test for those strains that were resistant to the drug. For the agar dilution method, ceftriaxone-sodium salt (Himedia, Mumbai, India) was used. Different dilutions of the antibiotics were used as per recommendations (10,11). ATCC *Escherichia coli* 25922 was inoculated on each plate as growth control. The E-test was performed as per the manufacturers’ instructions (Biomeriuex, India). Both the strains had an MIC value of >256 µg/mL for ceftiaxone. The ESBL detection was performed by combination disc method on the ceftriaxone-resistant isolates (10,12). These three strains, when phenotypically tested by the combination disc method, were positive for ESBL production. The DNA was extracted from the *Shigella* strains, using the boiling method (13). The DNA was subjected to PCR amplification targeting the known class of ESBL genes, using primers (Table 1) that would identify sequences encoding the ESBL genes TEM, CTX, and SHV.

The Multiplex PCR was done to identify *bla*SHV and *bla*CTX-M genes simultaneously (14). The reaction mixture consisted of 2 µL of DNA, 100 pmol (1 µL) concentration of each oligonucleotide primer of *bla*SHV, 30 pmol (1 µL) concentration of each oligonucleotide primer of *bla*CTX, master mix of 35 µL containing dNTPs, 1.25 U Taq polymerase and buffer with 1.5 mM MgCl_2_, and sterile nuclease-free water of 9 µL, making a total volume of 50 µL. The cycling conditions included an initial denaturation step at 94 ºC for 5 min and then 32 cycles with denaturation at 94 ºC for 45 sec, primer annealing at 50 ºC for 40 sec, and extension at 72 ºC for 60 sec. A final extension step at 72 ºC for 10 min was performed. The reaction mixture consisted of 4 µL of DNA, 2 µL of 100 pmol concentration of each oligonucleotide primer of *bla*TEM, master mix of 10 µL containing dNTPs, 1.25U Taq polymerase and buffer with 1.5 mM MgCl_2_, and sterile nuclease-free water of 7 µL, making a total volume of 25 µL. The cycling conditions used were 30 cycles of 94 °C for 1 min, 56 °C for 1 min, and 72 °C for 1 min, with a ﬁnal extension of 72 °C for 10 min (15). The resulting PCR products were documented on 1.5% agarose gel. PCR sequencing was performed to identify β-lactamase-resistant genes *bla*TEM, *bla*SHV, and *bla*CTX-M. Sequencing of β-lactamase gene amplicons was conducted by the Macrogen Inc., Seoul, Korea. The BLASTN program (www.ncbi.nlm.nih.gov/BLAST) was used for database searching. Using Multiplex PCR for *bla*CTX and *bla*SHV genes, 2 of the strains of *Shigella flexneri* were positive for *bla*CTX gene with a band-size of 544 bp. The sequencing results indicate that, out of both the strains harbouring the plasmid-encoded *bla*CTX genes, one matched the sequence of CTX-M-15 (with 100% identity, AC-FJ997866).

**Table 1.** Primers used in the study for detecting the ESBL genes

Primer	Sequence	Product-size (bp)	Reference
SHV-F	5'ATT TGT CGC TTC TTT ACT CGC-3’	1,018 bp	14
SHV-R	5’ TTT ATG GCG TTA CCT TTG ACC-3’		
CTXMU-1	5'ATG TGC AGY ACC AGT AAR GT 3’	544 bp	14
CTXMU-2	5’ TGG GTR AAR TAR GTS ACC AGA 3’		
TEM F	5'ATA AAA TTC TTG AAG ACG AAA 3’	1,076 bp	15
TEM R	5'GAC AGT TAC CAA TGC TTA ATC 3’		

Plasmid DNA extraction was done based on the principle of alkaline lysis method (16). The resulting plasmid DNA was separated in 0.8% agarose gels. The size of the plasmid was determined with a 500 bp ladder and a supercoiled plasmid DNA ladder, ranging from 500 bp to 33.5 kbp (Merck Specialities Private Limited, Bangalore, India). The plasmid profile of the plasmid DNA extracted from these isolates showed bands of 33.5 kbp.

Transformation experiments were performed using *E. coli* J53 strain as recipient previously described (17). For broth-mating studies, 0.1 mL of donors and 0.9 mL recipients (plasmid-free) were inoculated onto BHI broth culture made up to 10 mL volume and incubated at 37 °C for 18 hours. The recipient strain was plated onto LB agar plates supplemented with 6 µg/mL ceftriaxone as control. The transformed *E. coli* colonies were selected on LB agar plates and MacConkey agar plates supplemented with 6 μg/mL of ceftriaxone. Screening was carried out using the standard protocol (18). The transformed identities were reconfirmed based on their biochemical characters. The transformants were identified in the Blue–White screening agar, and their identities were confirmed with biochemical assays to be *E. coli*. Plasmid from the tranformant was extracted, which also showed the same plasmid as that of the donor strain (data not shown).

## DISCUSSION

Shigellosis is the major cause of dysentery in children. *S. flexneri* is the most predominating pathogen in developing countries, and the same is reflected in our study also. Antibiotics are indicated in the treatment for shigellosis. However, the alarming rise in antimicrobial resistance is a great threat to mankind. In the recent decades, majority of the members of the family Enterobacteriaceae were shown to produce extended-spectrum β-lactamase. This fact did not spare the genus *Shigella* where an SHV-11 ESBL-producing *S. dysenteriae* strain was reported in India in 1999 (19). There are several other reports of ESBL-producing *Shigella* from India (6-8). Third-generation cephalosporin-resistant *S. flexneri* isolate was first reported from a stool sample of a 16-month old child in Paris in 1995 (20). In the recent years, various ESBL-producing *Shigella* were also reported from Korea (CTX-M-14) (21), Argentina (CTX-M-2) (22), Viet Nam (CTX-M-15 and CTX-M-24) (23), and Turkey (24). In India, CTX-M-3 type of ESBL was reported in *S. sonnei* from Andaman and Nicobar islands (8). A novel CTX-M-64, a hybrid of CTX-M-15 and CTX-M-14, was reported by Nagano *et al*. from a shigellosis patient infected with *S. sonnei* after returning to Japan from China (25).

Conjugation experiments showed the transfer of the plasmid-encoded *bla*CTX-M-15 gene from the ESBL-producing *S. flexneri* isolate to *E. coli* J53. Transconjugants demonstrated resistance to third-generation cephalosporins mediated by transfer of a >35.5 kb plasmid. CTX-M-15 is a major CTX-M subtype which has spread all over the world and has been found in many members of the Enterobacteriaceae (26-28).

### Conclusions

Cephalosporins are the antibiotics of choice for severe and hospitalized cases, particularly in children where quinolones are not considered as treatment options by most clinicians. ESBL production in *Shigella* has complicated the situation, limiting the treatment options. Further, *Shigella* has the ability to carry multiple plasmids in relation to virulence and the antimicrobial resistance. In this study, we have described three *Shigella flexneri* strains which were resistant to ceftriaxone and were shown to produce ESBL by phenotypic and molecular methods. The major concern regarding these strains is that these were isolated from the stool samples of the children, in which case, most of the clinicians do not prefer using quinolones and cephalosporins. However, with the emergence of these strains showing resistance to the third-generation cephalosporins, the protocols for therapy of shigellosis need to be re-analyzed with emphasis on close continuous monitoring. This imparts that continued surveillance is needed to identify the ESBL-producers in patients with shigellosis which, in turn, will assist us in developing effective strategies in controlling the current situation.
